# The lower basal metabolic rate is associated with increased risk of osteosarcopenia in postmenopausal women

**DOI:** 10.1186/s12905-022-01754-6

**Published:** 2022-05-14

**Authors:** Zhila Maghbooli, Sadegh Mozaffari, Yasaman Dehghani, Pedram Rezaei Amirkiasar, Ali Asghar Malekhosseini, Mohamadtaher Rezanejad, Michael F. Holick

**Affiliations:** 1 Multiple Sclerosis Research Center, Neuroscience Institute, Tehran University of Medical Sciences, Tehran, Iran; 2 Department of Clinical Biochemistry, School of Medicine, Tehran University of Medical Sciences, Tehran, Iran; 3 Osteoporosis Research Center, Endocrinology and Metabolism Research Institute, Tehran University of Medical Sciences, Tehran, Iran; 4 School of Nursing and Midwifery, Shahid Sadoughi University of Medical Sciences, Yazd, Iran; 5 Vitamin D, Skin and Bone Research Laboratory, Section of Endocrinology, Diabetes, Nutrition and Weight Management, Department of Medicine, Boston University School of Medicine, Boston, MA USA

**Keywords:** Basal metabolic rate, Obesity, Osteoporosis, Sarcopenia, Osteosarcopenia

## Abstract

**Background:**

The goal of this study is to clarify clinical, functional, and biochemical features of postmenopausal women who are at risk of developing osteosarcopenia.

**Methods:**

This is a cross-sectional study undertaken to investigate the co-accordance of osteoporosis and sarcopenia and common risk factors on 305 postmenopausal Iranian women. Sarcopenia and osteoporosis were defined based on the European Working Group on sarcopenia in Older People guidelines and WHO criteria, respectively. Confounding factors including age, menopausal age, obesity, sun exposure, physical activity, macronutrient composition, and calcium and vitamin D supplementations were considered for all participants. A multivariate model was used to consider the common risk factors of both disorders; osteoporosis and sarcopenia.

**Results:**

The mean age was 57.9 years ± 6.0 SD (range: 48–78 years) and 37.4% of patients were 60 years or older. Among all participants, 35.7% were obese (BMI ≥ 30 kg/m^2^). Approximately 45% of all the study population had insufficient physical activity and at least half of participants had insufficient intake of protein. There was a significant correlation between bone density and muscle mass and basal metabolic rate (BMR) (*p* < 0.01). In multivariate-multivariable regression model, after Bonferroni correction for obesity, lower BMR was the only one associated with both lower muscle mass and bone density in lumbar and hip sites (*p* < 0.007).

**Conclusions:**

Our data suggest that low BMR might be an early predictor for concordance of osteoporosis and sarcopenia in postmenopausal women.

**Supplementary Information:**

The online version contains supplementary material available at 10.1186/s12905-022-01754-6.

## Background

Aging is raising principal health concerns as it predisposes to the risk of chronic disease among elderly people. Of these, osteoporosis and sarcopenia—two major musculoskeletal diseases—have been come into focus as these conditions are highly associated with increased morbidity and mortality worldwide, which poses a significant global health burden on the healthcare system.

Sarcopenia is described by attenuated muscle strength, loss of muscle mass, and decreased physical performance [1]. In this condition, a myriad of progressive age-related skeletal muscle loss alongside the disturbing muscular function (strength or physical performance) affects the routine daily activities, and escalates the risk of falls in particular [2]. In a population of postmenopausal women, the prevalence of sarcopenia ranges from 10 to 40% [3].

On the other hand, osteoporosis is a systemic bone disease that reduces bone microarchitecture and impedes bone strength which in turn leads to an increased risk of developing fractures even after subtle falls [2]. Osteoporosis affects roughly 30% of postmenopausal women who are above 50 years old [3].

However, a subset of older persons is affected by osteosarcopenia as a clinical condition in which the coexistence of osteoporosis and sarcopenia occurs. In this condition, the musculoskeletal system represents a trend towards low bone and muscle mass, while increased ectopic fat. This coexistence which is also stated as a “hazardous duet”, discriminates individuals at higher risk of falls, fractures, hospitalization, frailty, and mortality [4]. These negative consequences require proper detection and clinical care [5]. When the muscle strength and muscle mass start to evanesce, gradual loss of the ability to have an independent life appears and following affecting an individual turns out to be a troublesome social dilemma. Multi-morbidity is a prevalent incident that is observed in older people preponderantly. As the reason, disorders like heart failure or chronic obstructive pulmonary disease, expedite the loss of muscle mass and strength, thereby collaborating [6]. All of the points mentioned above, convey the concept of poor life quality burdened on the elderly patients [1].

A plethora of studies have demonstrated that a constellation of biochemical and genetic factors interacts with each other that increase the risk of osteoporosis and sarcopenia in elderly people. Specifically, bone and muscle could make an interaction by different anatomic, chemical, and metabolic aspects [2]. Previous studies have endorsed the presence of a communicative bridge over bone and muscle tissue which implies various paracrine and endocrine mechanisms to affect myokines and osteokines levels [7, 8]. These studies have also indicated a strong correlation between lean body mass (LBM) and bone mineral density (BMD) in women after menopause [7, 8].

Also, sarcopenia and increased fat mass can modify the energy metabolic rate and consequently basal metabolic rate (BMR) in postmenopausal women [9]. Several data point that a decrease in skeletal muscle reflected by the loss of LBM in patients with sarcopenia and an increase in adipose tissue cause a lower BMR [10, 11].

Recently, a tremendous effort has been made to identify the multiple factors that contribute to osteosarcopenia development in people with osteoporosis and osteopenia. However, uncertainty remains which risk factors could promote the coexistence of osteoporosis and sarcopenia that known osteosarcopenia in postmenopausal women. Here, we aimed to clarify clinical, functional, and biochemical features of postmenopausal women who are at risk of developing osteosarcopenia.

## Methods

### Study design and population

A cross-sectional study was undertaken to investigate the co-accordance of osteoporosis and sarcopenia and common risk factors on 305 postmenopausal Iranian women from the first of January to April 2016.

Participants were postmenopausal women who referred voluntarily undergone a routine health examination at the endocrinology clinic of Shariati hospital, Tehran, Iran. Written informed consent was obtained from all study participants in accordance with procedures approved by the Ethical Committee of Tehran University of Medical Sciences. Postmenopausal women with serious and/or chronic illnesses, especially those with diabetes mellitus, heart, liver, and kidney disorders and use of steroidal drugs (prednisolone, dexamethasone, and betamethasone), use of hormone therapy within the last 6 mounts, or use of antiosteoporotic drugs (raloxifene and bisphosphonates) in the last 2 years were not eligible to participate in the study. Only five women had a history of smoking that were excluded of data analyses. None of participants had a history of alcohol consumption.

### Data collection

Demographic and clinical information including age, menopausal age, and current use of medications and vitamin supplementations within the last 6 months were obtained by a questionnaire. Anthropometric measurements of the participants were taken using standard anthropometric techniques. Body mass index (BMI) was calculated as body weight (in kilograms) divided by height (in meters) squared. Obesity was defined as having BMI equal to/or more than 30 kg/m^2^ [12].

### Bone status measurements

Dual X-ray absorptiometry with a Lunar DPXMD densitometer (Lunar 7164, GE, and Madison, WI, USA) was used to assess the BMD in two bone sites: Total hip, and spinal lumbar vertebras (L2–L4). Precision error in BMD measurements was 1–1.5% in the lumbar and 2–2.5% in the hip regions. Each person was categorized based on the WHO osteoporosis criteria: Osteoporosis (T score ≤  − 2.5), osteopenia (− 2.5 < T score <  − 1) and normal (T score ≥  − 1).

### Sarcopenia definition

To identify sarcopenia, we used the EWGSOP (European Working Group on Sarcopenia in Older People) criteria (2018) [2]. Probable sarcopenia is identified by low muscle strength. The diagnosis of sarcopenia is confirmed by low muscle strength plus low muscle mass.

Participants who only have low muscle mass were classified as presarcopenia; those who have low muscle mass plus low muscle strength, or low physical performance were as sarcopenia.

#### Muscle strength

Muscle strength was assessed by handgrip strength, which was measured with a dynamometer (Jamar dynamometer). Three trials for each hand were performed and the average measurements of both hands were used in the analysis. Using the cut-off points suggested for different- sex–age groups less than 19.7 kg for women.

#### Muscle mass

Muscle mass was estimated by Bioelectrical impedance analysis (BIA) resistance (Tanita BC-418 manufactured by Tanita Corporation, Tokyo, Japan). The appendicular muscle mass index (ASMI) was calculated by the following equation: skeletal muscle mass (kg) divided by the square of height (m^2^). To assess reproducibility, whole-body DEXA was used for 20% of participants for muscle mass assessment with Cronbach's Alpha equal 0.92. Percentage Fat Mass (PFM) (the ratio of fat mass to body weight) and basal metabolic rate (BMR) (Kcal/24 h) were estimated by the same BIA equipment. Low muscle mass was defined as an ASMI ≤ 5.7 kg/m^2^.

#### Physical performance

Usual walking speed on a 4-m course was evaluated by measuring subjects’ gait speed (meter/second). The cut-off ≤ 0.8 m/s was classified as of low physical performance. Nobody had a history of disability of walking.

### Biochemical measurements

Blood samples were taken after at least 10 h of overnight fasting. The separate sera were kept at − 80 °C until analysis. 25(OH)D was measured using an electrochemiluminescence assay (ELECSYS^®^ Vitamin D assay, Roche, Germany). A cut-off value of 30 ng/mL was used for optimal vitamin D status; vitamin D deficiency/insufficiency was defined as 25(OH)D less than 30 ng/mL [12, 13].

### Dietary assessments

Dietary intakes were assessed by a validated semi-quantitative 146-item food frequency questionnaire (FFQ) [14]. Participants were asked to report their usual frequency of consumption for each FFQ item and also the amounts of intakes based on the serving sizes which are noted in the FFQ over the past year. Dietary intakes of each food were then converted to grams per day and the nutrient contents of food items (Macronutrients) were calculated according to the USDA food composition databases. Macro nutrition percentages were calculated based on total energy.

### Sun exposure

For sun exposure, a questionnaire was completed including 4 questions; time per day, day time, an average time of sun exposure, and using sunscreen during the last three months. Sun exposure was classified based on at least 10 min per day at daytime (between 10 am to 3 pm).

### Physical activity

The short format of the International Physical Activity Questionnaire (IPAQ) was used to assess physical activity [15]. Following IPAQ’s guidelines, frequency and duration of physical activity were converted to Metabolic Equivalent of Tasks (MET). Physical activity was classified into three levels: inactive, moderate activity, and health- enhancing physical activity (HEPA).

### Statistical analysis

Data were analyzed by SPSS statistical software SPSS^®^ 20 (IBM Corporation, Armonk, NY, USA). ANOVA and post hoc tests and Chi-square test were applied to consider the difference between confounding factors in women with osteoporosis, osteopenia, and normal bone density. A Pearson’s correlation was used to determine the correlation between bone density and muscle mass and risk factors including age, menopausal age, BMI, and macronutrients.

Univariate linear regression model was used to consider the correlation between confounding factors and bone and muscle status. A multivariate-multiple linear regression model was used to consider the common risk factors of both disorders. The dependent factors were the bone density in lumbar and/or hip sites (T-score), and the appendicular muscle mass index (ASMI). The covariates were age, menopausal age, macronutrients, and BMR. The fix factors were sun exposure, physical activity, taking calcium and vitamin D supplementations and obesity. If *p* value was less than 0.2 (*p* value < 0.2), the cofactor was adjusted in the multivariate model.

Numerical variables were expressed as the mean ± standard error (SE), and categorical variables were presented as percentages. Two-tailed *p* values less than 0.05 were considered significant.

## Results

A total of 305 postmenopausal Iranian women were enrolled in this study. The mean age was 57.9 years ± 6.0 SD (range: 48–78 years) and 37.4% of patients were 60 years or older. Bone status and muscle mass were considered for all women.

The baseline characteristics of all participants are presented in Table 1. Confounding factors including obesity, sun exposure, physical activity, macronutrient composition and calcium, and vitamin D supplementations were considered for all participants (Table 1). Among all participants, 35.7% were obese (BMI ≥ 30 kg/m^2^). Approximately half of all study population had insufficient physical activity (44.7%). Macronutrients including carbohydrates, fat, and protein were calculated based on total energy. Among all participants, the range of protein intake was 9–24% and 58.8% of women had insufficient intake of protein; less or equal to 15%.

**Table 1:** Demographic characteristics and biochemical analyses and skeletal-muscle status of the study population

Demographic characteristic	N	Total	Min	Max
Age (year)^±^	305	58 ± 6	46.0	78.0
Menarche age (year)^±^	305	13 ± 1	9	18
Menopause age (year)^±^	305	48 ± 5	34.0	62.0
Gravity^±^	305	4 ± 2	0	12
Parity^±^	305	3 ± 2	0	10
Lactation (month)^±±^	305	36 (45)	0	192
BMI (kg/m^2^)^±^	305	28.6 ± 4.6	17.5	41.9
Physical activity	292			
Insufficiently activity		44.9% (131)		
Minimally activity		36.3% (106)		
HEPA activity		18.8% (55)		
Vitamin D supplementation	305	25.5% (78)		
Calcium supplementation	305	45.2% (138)		
Sun exposure (equal or more than 10 min/10 am to 3 pm)	301	40.9% (123)		
Sun screen (using usually or always)	301	41.5% (125)		
*Bone status*	305			
Osteoporosis		21.0% (64)		
Osteopenia		51.8% (158)		
Normal		27.2% (83)		
*Muscle status*				
Appendicular muscle mass index (ASM/hight^2^)^±^	305	7.1 ± 0.9	4.43	9.37
Low (≤ 5.76)		9.2% (28)		
Moderate (5.76–6.76)		23.9% (73)		
Normal (≥ 6.76)		66.9% (204)		
Handgrip strength (Muscle strength) < 19.7 kg	271	43.9%(108)		
Gait speed (Physical performance) ≤ 0.8 m/s	216	19.9% (43)		
*Macro-nutrients*				
Total protein (Percentage of energy)^±^	305	15 ± 2	9	24
Total carbohydrate (Percentage of energy)^±^	305	61 ± 5	39	74
Total fat (Percentage of energy)^±^	305	28 ± 5	18	67
Total energy intake (kcal)^±^	305	2173 ± 425	1171	3618
*Body composition*				
Fat (%)^±^	305	37 ± 6	14	60
BMR (kcal)^±^	305	1320 ± 132	948	1744
FFM (kg)^±^	305	42 ± 7	10	70

Numerical variables were expressed as the mean ± SD and categorical variables were presented as percentages

*BMI* body mass index, *BMR* basal metabolism rate, *FFM* free fat mass, *HEPA *health- enhancing physical activity

N = available data for each variable, Min = minimum, Max = maximum, Ln = natural logarithm

^±^Mean ± SD

^±±^Median (IQR)

Circulating serum concentrations of 25(OH) D were measured in 195 women with mean 37.7 ng/ml ± 18.8 SD (range: 3.2–79.9 ng/ml). A cutoff point less than 30 ng/mL of 25(OH)D was used for the definition of vitamin D deficiency/insufficiency. In total, 36.4% of the patients had a 25(OH)D level of less than 30 ng/ml.

### Bone status

Among all participants, 21.0% (83) had osteoporosis with a T-score of less than − 2.5 in at least one region (total hip, or lumbar); 13.11% (40) with a T-score of equal or less than − 3. Based on osteoporosis classification, the baseline characteristics of participants are summarized in Additional file 1: Table S1.

### Muscle status

Based on EWGSOP criteria (2018), about 40% of women were diagnosed as having probable sarcopenia (weak muscle strength), and 15.1% suffered from both low muscle mass and weak muscle strength.

### Co-accordance risk of osteoporosis and sarcopenia (osteosarcopenia) in postmenopausal women

In Pearson’s correlation analysis, there was a significant positive correlation between bone density (T-score hip, and T-score L2–L4) and ASMI (*p* < 0.01) (Table 2). There was a significant correlation between bone density and muscle mass and age, BMR, and BMI (*p* < 0.01). There were no significant correlations between bone density and muscle mass with menopausal age in our study population. There was no significant correlation between bone density and ASMI with 25(OH) D (Table 2).

**Table 2:** The relationship between bone and muscle mass status and osteosarcopenia risk factors

	ASMI	Age	Menopausal age	BMR	BMI	25(OH) D
Hip T-score	0.2**	− 0.2**	0.01	0.3**	0.2**	− 0.1
Lumbar L2–L4 T-score	0.1**	− 0.2**	0.009	0.2**	0.1*	− 0.1
ASMI	1	− 0.009	− 0.07	0.7**	0.8**	− 0.1

*ASMI* appendicular muscle mass index, *BMR* basal metabolism rate, *BMI* body mass index, *FFM* free fat mass

Boldface indicates statistical significance (*p* value < 0.05)

*p Value < 0.01

**p Value < 0.0001

To address the risk factors associated with osteosarcopenia in our population study, a univariate model was used to assess the association between risk factors and bone density (Hip T-score and Lumbar T-score) and muscle mass (ASMI), separately. In the univariate model, after adjusting confounding factors [age, obesity, physical activity, calcium and vitamin D supplementations (*p* < 0.2)], BMR had an independently significant association with T-score of the hip (*p* = 0.0001) and T-score on the lumbar L2–L4 (*p* = 0.02). A similar analysis was performed for considering muscle mass risk factors. After adjusting confounding factors [age, obesity, physical activity, and percentage of protein consumption (*p* value < 0.2)], BMR had independently a significant association with muscle mass (*p* = 0.0001).

In multivariate model, after Bonferroni correction for obesity, only lower BMR was associated with both lower muscle mass and bone density in lumbar (*p* < 0.006) and/or hip sites (*p* = 0.0001)with adjusted R-square = 0.56.

## Discussion

There is ample evidence that sarcopenia and osteoporosis share common risk factors and also share the same biological pathways [4]. In our current study, we showed that lower BMR was independently associated with both lower muscle mass and bone density in lumbar and hip sites. In line with our results, Soysal et al. had reported a linkage between a lower BMR and the upcoming emergence of sarcopenia and frailty in elderly men. The authors mentioned the ratio of BMR to body surface area, which used to contradict the effect of height and weight on BMR, could be expressed as an objective feasible screening method for sarcopenia [16]. In another study, it was suggested that BMR might be a predictor for osteoporosis as they found that BMR and body fat significantly predicted BMD of the femoral neck and vertebral BMD among women over 50 years of age [17].

Once humans take steps into senescence, conditions such as reduced total energy expenditure and energy availability start to appear [16, 18]. More importantly, energy metabolism and body composition are highly interrelated; a higher basal metabolic rate reflects the metabolically active tissue [18, 19], and the skeletal muscle tissue is stated to consume the highest amount of energy needed to meet the entire human body requirements [16].

Although it was beyond our scope to determine how decreased BMR can be considered as a possible risk factor associated with osteosarcopenia, several possibilities can be considered. By modeling, we tried to minimize the effect of main confounding factors including age, menopausal age, obesity, physical activity, macronutrient composition, and vitamin D intake, but they may modify the association between lower BMR and risk of osteosarcopenia through different cellular mechanisms. Evidence points to a decrease in BMR with advancing age. BMR is the energy expended by a person at rest and accounts for approximately 60–75% of daily energy expenditure in individuals with a sedentary lifestyle. Reduced energy consumption is not only emerged by declined BMR but also observed alongside the decreased intensity and duration of physical activities. Additionally, reduced fat oxidation might give rise to the diminution of postprandial energy expenditure [16, 19]. Moreover, BMR tends to increase with inflammatory conditions or age-related chronic diseases [16, 20].

Fat-free mass (FFM) is the most significant factor which determines BMR, and age is also known as one of the most important regulators of energy metabolism. In fact, skeletal muscle loss in sarcopenia results in BMR reduction by roughly 30% between the ages of 20 and 70 years [16]. Noticeably, in our study, BMR also had a significant positive correlation with FFM and a negative correlation with age.

Although the protein synthesis in the skeletal muscle is influenced by its degradation, bone formation is modulated by its resorption. An imbalanced adjustment of the mentioned mechanisms will give rise to low BMD and/or sarcopenia. Both conditions tend to be multifactorial and also have various pathophysiological pathways in common [2]. Among our population study, the range of protein intake in postmenopausal women was 9–24% and over half of participants had less or equal 15% protein intake.

In our study obesity was a common risk factor for both osteoporosis and sarcopenia. The emerging evidence shows that fat tissue influence the skeleton system. There is a report that fat tissue exerts its protective effects through applying its buffering characteristics which protect the bones from fragility. Several studies indicated that a higher BMI represents a protective factor against the presence of osteosarcopenia [1, 21–27]. While other investigations have clearly shown that high BMI and low body mass have deleterious effects on the disease progression leading to the increased mechanical pressure on bones and the diminution of muscular support as well [28]. Fatty infiltration of bone and muscle is mostly related to the negative effects caused by the secretion of inflammatory cytokines from bone marrow and body fat in an especial process called lipotoxicity [29]. Several endocrine factors such as insulin-like growth factor-1, osteoglycin, irisin, osteonectin, fibroblast growth factor-2, IL-6, IL-15, and myostatin are released from muscle and affect bones subsequently [30].

Concomitant to the onset of the aging process, diminution of physical activity overshadows the entire elderly lifestyle resulting in the substantial increase in the time spent sedentarily [8, 31], thereby the conditions like mechanical loading loss and bone and muscle wreckage are likely to appear subsequently [8, 32]. In our study population, around 80% of women had insufficiency/minimum activity. Because sun exposure declines by around 25% between the ages of 40 and 70 years [8, 33], this results in decreased vitamin D production and lower circulating levels of 25(OH)D, putting postmenopausal women at risk of muscle weakness [8, 34] as well as bone loss. Additionally, deficient levels of vitamin D underlie the augmented risk of falls through its multiple effects on muscle and bone [8, 35]. In our study, although 36.4% of the women had vitamin D deficiency/insufficiency, there was not any significant correlation between bone and muscle mass status with circulating levels of vitamin D. However, measuring vitamin D levels in the blood at a single point in time may not reflect a person’s true vitamin D status.

Some limitations in our study are worth noting. Firstly, postmenopausal women are at risk of osteoporosis, while people older than 65 are more at risk of sarcopenia. However, to identify individuals who are likely to be at risk of development of both osteoporosis and sarcopenia, we considered postmenopausal women. Occurrence of BMD loss accompanied by gradually declining muscle mass, strength, and function concomitantly has been introduced to identify risk factors sarcopenia in women with osteopenia/osteoporosis. Secondly, it was documented that smoking increases the risk of developing sarcopenia. In this consent, a recent cohort study considered the role of smoking on the risk of sarcopenia [36]. They mentioned that “smokers have a 2.36-fold higher risk of developing sarcopenia”. In our data, only 5 women had a history of smoking that were excluded of the analysis. Lastly, the design of our study is cross-sectional. So, we cannot explain the cause and effect relationship.

## Conclusions

In summary, the present study along with others provides evidence that lower BMR was associated with both lower appendicular muscle mass and bone density in the lumbar spine and hip. Since the current study was a cross-sectional design, long-term prospective cohort studies are needed to monitor the occurrence of osteosarcopenia in postmenopausal women.

## Supplementary Information

**Additional file 1:**Characteristics, biochemical and environmental factors based on osteoporosis classification.

## Data Availability

The data generated during and/or analysed during the current study are available from the corresponding author on reasonable request.

## Abbreviations

ASMI: Appendicular muscle mass index
BIA: Bioelectrical impedance analysis
BMD: Bone mineral density
BMI: Body mass index
FFQ: Food frequency questionnaire
IPAQ: Physical activity questionnaire
MET: Metabolic equivalent of tasks
PTH: Intact parathyroid hormone
