# Correction: The lower basal metabolic rate is associated with increased risk of osteosarcopenia in postmenopausal women

**DOI:** 10.1186/s12905-022-01913-9

**Published:** 2022-08-01

**Authors:** Zhila Maghbooli, Sadegh Mozaffari, Yasaman Dehhaghi, Pedram Rezaei Amirkiasar, Ali Asghar Malekhosseini, Mohamadtaher Rezanejad, Michael F. Holick

**Affiliations:** 1 Multiple Sclerosis Research Center, Neuroscience Institute, Tehran University of Medical Sciences, Tehran, Iran; 2 Department of Clinical Biochemistry, School of Medicine, Tehran University of Medical Sciences, Tehran, Iran; 3 Osteoporosis Research Center, Endocrinology and Metabolism Research Institute, Tehran University of Medical Sciences, Tehran, Iran; 4 School of Nursing and Midwifery, Shahid Sadoughi University of Medical Sciences, Yazd, Iran; 5 Vitamin D, Skin and Bone Research Laboratory, Section of Endocrinology, Diabetes, Nutrition and Weight Management, Department of Medicine, Boston University School of Medicine, Boston, MA USA

## Correction: BMC Women’s Health (2022) 22:171 https://doi.org/10.1186/s12905-022-01754-6

Following the publication of the original article [1], the author name ‘Yasaman Dehhaghi’ has been misspelled as ‘Yasaman Dehghani’.

The original article has been corrected.
