# Supplementary material for: The lower basal metabolic rate is associated with increased risk of osteosarcopenia in postmenopausal women
Source: BMC Womens Health. 2022 May 14;22:171. doi: 10.1186/s12905-022-01754-6 (PMC9107118; doi:10.1186/s12905-022-01754-6)
Supplement: Supplementary file 1 — Additional file 1:Characteristics, biochemical and environmental factors based on osteoporosis classification. [file 12905_2022_1754_MOESM1_ESM.docx]

**Characteristics, biochemical and environmental factors** **based on osteoporosis classification**

The baseline characteristics, biochemical and environmental factors of all participants are shown in **Table S1.** The data were presented based on osteoporosis classification.

The data showed there were significant differences in age among groups (p<0.5). Post hoc test showed there were not significant differences in age between women with osteopenia and control group. While, women in osteoporosis group were tended to be older and compared with osteopenia and control groups (p<0.05). Further analysis of the data showed no differences in menopausal age, obesity, physical activity among groups (p>0.05). Taking vitamin D supplementation was less in control group, but the differences were not statistically significant among groups (p=0.12).

The circulating levels of vitamin D were lower in control group, but the differences were not statistically significant (p=0.08). Based on vitamin D classification, vitamin D deficiency was higher in control group (47.6%) than osteopenia (36.4%) and osteoporosis (14.7%) groups (p=0.01).

Regarding bone markers, there were not significant differences in the circulating levels of CTX, OC, and PTH among osteoporosis, osteopenia and control groups (p>0.05).

**Table S1:** Descriptive and biochemical characteristics of the study population based on osteoporosis classification

|  | **N** | **Normal (N=83)** | **Osteopenia (N=158)** | **Osteoporosis (N=64)** | **P-value** |
| --- | --- | --- | --- | --- | --- |
| Age | 305 | 56.3±5.5^a^ | 57.8±6.2^b^ | 60.09±5.7^ab^ | .001 |
| Menarche age (year) | 305 | 13.2±1.3 | 13.4±1.2 | 13.6±1.6 | .2 |
| Menopause age (year) | 305 | 48.8±5.5 | 48.1±4.3 | 47.9±4.8 | .4 |
| Gravity | 305 | 3.4±1.6 | 3.8±2.1 | 4.1±2.6 | .2 |
| Parity | 305 | 2.8±1.3 | 3.1±1.8 | 3.3±2.1 | .2 |
| Lactation (month) | 305 | 38.4±29.3 | 44.7±42.5 | 42.7±40.6 | .5 |
| BMI (kg/m^2^) | 305 | 29.5±4.4 | 28.5±4.7 | 27.9±4.4 | .1 |
| Physical activity | 305 |  |  |  |  |
| Insufficiently active |  | 35.4% (29) | 49.7% (74) | 45.9% (28) | .1 |
| Minimally activity |  | 45.1% (37) | 34.2% (51) | 29.5% (18) |  |
| HEPA activity |  | 19.5% (16) | 16.1% (24) | 24.6% (15) |  |
| Vitamin D supplement | 305 | 22.9% (19) | 23.4% (37) | 34.4% (22) | .2 |
| Calcium supplement | 305 | 53.0% (44) | 43.0% (68) | 40.6% (26) | .2 |
| Sun exposure (equal or more than 10min/10 am to 3pm) | 301 | 42.7% (35/82) | 37.8% (59/156) | 46.0% (29/63) | .5 |
| Sun screen (using more than usually) | 301 | 47.6% (39/82) | 41.0% (64/156) | 34.9% (22/63) | .3 |
| BMR (Kcal) | 305 | 1351±124.5^a^ | 1320.8±135.64^b^ | 1274.9±122.7^ab^ | .002 |
| FAT (%) | 305 | 37.6±5.1^a^ | 37.2±6.2 | 35.19±6.5^a^ | .03 |
| FFM (kg) | 305 | 43.8±6.3^a^ | 42.3±7.0 | 40.51±7.0^a^ | .01 |
| ASMI (kg/hight^2^) | 305 | 7.2±0.9^a^ | 7.1±0.9 | 6.91±0.9^a^ | .1 |

Appendicular muscle mass index (ASMI), Basal metabolism rate (BMR), Body mass index (BMI), Free fat mass (FFM).

Numerical variables were expressed as the mean ± SD and categorical variables were presented as percentages.

ANOVA test for numerical variables and Pearson Chi-Square test for categorical variables

Significant Post Hoc test (Tukey); a: comparison between Normal and Osteoporosis groups, b: comparison between Osteopenia and Osteoporosis groups

N=available data for each variable
